# Editorial: Positive youth development, mental health, and psychological well-being in diverse youth

**DOI:** 10.3389/fpsyg.2023.1152175

**Published:** 2023-05-30

**Authors:** Nora Wiium, Laura Ferrer-Wreder, Jennifer E. Lansford, Lene Arnett Jensen

**Affiliations:** ^1^Department of Psychosocial Science, University of Bergen, Bergen, Norway; ^2^Department of Psychology, Stockholm University, Stockholm, Sweden; ^3^Sanford School of Public Policy, Duke University, Durham, NC, United States; ^4^Department of Psychology, Clark University, Worcester, MA, United States

**Keywords:** positive youth development, mental health, psychological well-being, diverse contexts, global

## 1. Introduction

This is an editorial commentary on the Research Topic entitled: Editorial: Positive youth development, mental health, and psychological well-being in diverse youth. One impetus behind this Research Topic was to examine the ways in which positive development intersects with problematic aspects of development and behavior, with an emphasis on connections to indicators of mental health problems. The second impetus was to take an international approach, including youth from diverse countries and settings who were studied using a variety of etic and emic methods that correspondingly consider cross-culturally common features and features that vary across cultures (Lansford et al., 2021).

We and the authors of the present articles approached these two goals with the knowledge that positive youth development (PYD), in some cases also called youth development (Roth and Brooks-Gunn, 2016), takes many forms (e.g., Benson, 2007; Geldhof et al., 2014). Several theoretical models are relevant to explaining PYD (Wiium and Dimitrova, 2019; Dimitrova and Wiium, 2021). Many of these theories posit that one should expect adaptation to occur when individual and contextual resources are in synergy with one another, and that these points of mutuality between person and context lead to new opportunities for development and growth for individuals and their contexts (Lerner et al., 2017).

Theories of PYD have proposed that an adaptive alignment of personal strengths (i.e., competencies, skills, and self-perception) with contextual resources (i.e., relational, and other environmental resources and opportunities) will facilitate thriving, which is expressed as young people contributing to themselves, others, and their communities (Geldhof et al., 2014; Lerner et al., 2017). Although one would expect that more strengths would imply fewer problems, the PYD field is also currently empirically examining a more nuanced expectation about the range of possible intersections between individual strengths and problems. Diverse youth may differ in how their strengths are related to problems, such as anxiety, depression, substance use, and other risky behaviors. Initial empirical findings suggest that there may indeed be a complex connection between strengths and problems for some groups of children and youth (e.g., Arbeit et al., 2014; Kozina et al.), whereas for other children and youth, strengths and problems are simply inversely related (i.e., more strengths in the presence of fewer problems and vice versa). Understanding the nuanced connections between strengths and problems is clearly being advanced by diversity in measurement that is ecologically salient as well as diverse approaches to understanding data, including the use of both variable- and person-oriented approaches (Johnson and Ettekal, 2022).

Despite the possibly complex associations between strengths and problems, a strong deficit focus has historically been prevalent in the study of child and adolescent development. Moreover, the focus on deficits has been especially pronounced in research with underrepresented cultural groups (Coll et al., 1996; Vélez-Agosto et al., 2017). A more holistic approach to development involves a consideration of both strengths and problems. Also crucial is a fulsome consideration of the diversity of cultures and contexts in which development takes place (Jensen, 2016).

The present Research Topic advances the field by adding new empirical evidence about possible complex associations between strengths and problems and by adopting an ecological approach attuned to the importance of the multiple cultures and contexts of development in which children and young people live and grow. The article collection in our Research Topic includes research articles from the Cross-National Project on Positive Youth Development initiated at the University of Bergen in 2014 (Wiium and Dimitrova, 2019), and numerous contributions from other researchers from around the globe. Together, these studies highlight the application of strengths-based approaches in many cultures and contexts, some of which have had very little or no research attention on youth development.

Specifically, the present 30 articles included children, adolescents, and emerging adults from many different countries in Africa, the Americas, Asia, and Europe. The researchers conducting the work also spanned these continents. The inclusion of culturally diverse participants and researchers provides for a more valid developmental science (Jensen, 2016; Jensen and Arnett, 2020). Here, the findings from participants and the perspectives of the researchers provide new insights into how PYD involves both cross-culturally common features and features that vary across cultures. Understanding both what is culturally common and distinctive about PYD is important for the sake of scientific knowledge and for applied purposes. Not only will the application of findings be more successful when based upon knowledge that is ecologically valid to the cultural backgrounds of the youth in question, but such knowledge is also increasingly important as youth and cultures are ever more connected in a globalizing world (Jensen, 2021).

## 2. Highlights from the article collection

Published in different article formats, namely meta-analysis, study protocols, book reviews, and empirical papers of varying research designs such as cross-sectional, longitudinal, and intervention studies, the 30 articles in this Research Topic highlight the individual and contextual factors that can have implications for the mental health and psychological well-being of children, adolescents, and emerging adults living in a wide variety of countries. In this section, we offer highlights of these 30 articles^1^ to provide a sense of their themes and novel contributions.

### 2.1. Articles advancing the understanding of positive development in its own right with perspectives from various parts of the globe

Several contributions in this area span different article types as well as varied developmental periods. For example, Hinerman et al. reported on the psychometric utility of a scale of social emotional and character development with a sample of children (aged 10 to 13 years old) living in Belize. Fan and Fan in a moderated mediation model presented findings that indicated the significant role of basic psychological need satisfaction and resilience in the association of social support from family, friends, teachers, and other significant contexts with psychological adjustment among Chinese left-behind rural adolescents (aged 8 to 17 years old).

In their protocol research paper, Ng et al. proposed a theoretical model of the role of empathy, parental differential treatment, and fairness on psychosocial well-being among secondary school students in Hong Kong. Bowers et al. examined nature as an ecological asset for thriving among 11- to 14-year-old middle school students living in low-income communities in the U.S. and observed that connection to nature as well as time in nature was related to an overall thriving score reflecting the 5Cs (competence, confidence, connection, character, and caring or compassion) of PYD. In another U.S. study comprising youth of color (aged 11 to 18 years old) attending an afterschool college preparation program, Bowers et al. results indicated that among Latinx youth, contribution (e.g., helping others and volunteering) was predicted by the youth's critical reflection, hopeful future expectations, and the relationship they had with an adult mentor in the program.

Su and Liu cross-temporal meta-analysis of studies involving 100 studies and 55,830 Chinese college students indicated temporal changes in cohorts' subjective well-being with a consideration of temporal changes in social indicators. Further, in a different study involving 17- to 25-year-old Chinese college students, Jiang et al. results indicated that coping style is important for the students' subjective well-being. The authors found that positive coping is indirectly related to life satisfaction and expression suppression through cognitive reappraisal and positive affect, respectively. Fernandes et al. study with 15- to 25-year-olds living in Kosovo, Norway, Portugal, Slovenia, Ghana, and Turkey found unique patterns of strengths using different theoretical views of PYD.

### 2.2. New empirical findings about connections between strengths and problems

Several of the empirical papers included in this Research Topic investigated the question of strengths and problems. For example, Novak et al. in a sample of Croatian adolescents (aged 14 to 19 years old) found several inverse associations between greater school- and family-related factors (e.g., school attachment and commitment, family communication and satisfaction) with lower reported depression, anxiety, and stress. However, there was moderation of some of these findings by gender and positive associations between strengths and problems for some particular constructs. For another sample of Croatian adolescents (with mean age of 16.78), Kurtovic et al. found that individual-level factors like youth contribution to family, friends, school, and community along with self-regulation and academic performance were related to lower levels of depression symptoms. Self-regulation and academic performance were also found to be important mediators of the association between contribution and depression. Wang et al. study on the importance of micro- and macro-level factors revealed the mediating and moderating roles of PYD attributes (e.g., bonding and resilience) and migrant status on the association between family functioning and internalizing problems like anxiety and depression among Chinese adolescents (aged 12 to 18 years old).

In a cross-national study involving three European countries, Kozina et al. reported significant findings on the differential role of the 5Cs of PYD on anxiety among children, youth, and emerging adults between the ages of 10 and 29. Gomez-Baya et al. found in samples of high school and university students (with mean age of 19.37) from Eastern Croatia and Southern Spain that the psychological strengths of confidence and connection were negatively associated with depressive symptoms. In a longitudinal study of first year university students in Lithuania (aged 18 to 29 years old at T1), Truskauskaite-Kuneviciene et al. investigated how identity profiles were related to traumatic experiences. The authors identified three distinct identity profiles: diffused, undifferentiated, and coherent identity statuses, but did not find any association with lifetime trauma exposure. Zhou et al. findings on Chinese adolescents (with mean age of 13.12) revealed concurrent and longitudinal associations of PYD attributes with life satisfaction and hopelessness, along with hopelessness also mediating the longitudinal association between PYD attributes and life satisfaction. In the Latin American context, Manrique-Millones et al. observed inverse associations between developmental assets (reflecting personal strengths and contextual resources at home, school, and community) and substance use, as well as positive associations between the assets and the social, emotional, and psychological well-being of college students in Colombia and Peru (aged 17 to 30 years old).

### 2.3. Contributions that inform intervention science

In a protocol research paper, Ungar et al. explained how using a mixed method approach in conducting multi-systemic resilience research can be an example of how data analyzed from multiple systems can inform socially and contextually relevant interventions and policies regarding youth resilience. In the area of children's social emotional competence, Eninger et al. reported on the results of the first test of the preschool edition of Promoting Alternative Thinking Strategies (PATHS^®^) with Swedish children (4- to 6-year-olds). Further, several of the studies add new information about the associations between strengths and problems that highlight their independence in some cases and the need for interventions to ameliorate problems not only through a consideration of risk but also strengths and resources, even in less-than-optimal circumstances (Catalano et al., 2002). Some preschool aged social emotional learning interventions that have been well implemented (e.g., Watts et al., 2018; Bierman et al., 2021) have been associated with long-term beneficial outcomes in adolescence, and thereby illustrate the vital importance and value added to work in all developmental periods to further support resources within children and youth as well as in key contexts of development (e.g., schools, at home, in neighborhoods) and at key turning points of developmental and during social transitions.

## 3. Conclusions about the 30 articles

The 30 articles in this Research Topic contribute to the PYD perspective in varying ways. Theoretically, the collection lends support to the assumption that youth development when considered from a positive perspective is diverse in expression and in processes of development. In general, most of the evidence found that personal strengths, resources, and opportunities in youth contexts are likely to be inversely associated with risk and problem behaviors for many children but that there is a need to understand these associations in their totality with the expectation that there can be exceptions and nuances to this overall connection. The PYD assumption in relation to developmental systems models prescribes an optimal alignment between youth strengths and contextual resources as the condition necessary for positive development to occur. The empirical evidence from the articles in the collection largely supports the PYD theoretical assumption. However, the optimal alignment recommendation is difficult to examine in solely nomothetic research approaches. Measurements at both individual and contextual levels are not always achieved in developmental science in general or in the present studies, suggesting that more work needs to be done regarding the application of the PYD perspective in research.

Besides the findings on the critical role of the ecology of children, adolescents, and emerging adults on their developmental outcomes, the article collection provides a broader global perspective of PYD and strengths-based approaches, with its inclusion of cultures and contexts of the majority world and the unheard voices of youth in diverse settings. Together, these findings extend the empirical evidence of the personal strengths and contextual resources implicated in the prominent PYD frameworks, but also confirm the influence of other micro- and macro-level factors that are not readily captured in these theoretical frameworks. Thus, the findings from the article collection highlight the important role of skills, competencies, resources, and opportunities in the ecology of young people from diverse backgrounds.

## 4. Looking forward: advancing the PYD field

International collaborations can advance understanding of PYD by combining emic and etic perspectives of cultural insiders and cultural outsiders, respectively (e.g., Lansford et al., 2021). When studying individuals who share their own cultural background, researchers are often better able to understand indicators of supportive environments and positive adjustment that may not be apparent to cultural outsiders (Chai et al., 2022). Emic approaches also facilitate understanding of novel aspects of positive development that may be important in a given context but that would be missed if they were not incorporated in theories and assessments developed elsewhere (McWayne et al., 2017). Recognizing strengths of young people, families, and communities rather than adopting a deficit perspective makes it more likely that researchers will understand how and why certain cognitions and behaviors are adaptive in particular cultural contexts. International collaborations make it possible to develop theories that extend beyond those limited by their typical origins in Western, high-income countries and to test the replicability and generalizability of findings across diverse cultural groups, an important goal in contemporary psychological science (e.g., Milfont and Klein, 2018). However, international collaborations also present challenges, including decisions about whether to adapt existing measures to make them appropriate for use in new contexts or to generate new measures, particularly to avoid being “underinclusive” of the construct of PYD by missing indicators that are important in some but not other contexts (Van de Vijver, 2017).

Exclusively emic approaches have the disadvantage of making it more difficult to compare how individuals in different contexts fare in relation to one another, which can be important to motivating policy action and mobilizing resources to support PYD. For example, the Sustainable Development Goals guiding the international agenda through 2030 outline a number of areas that are essential to PYD, such as eliminating poverty (Goal 1), promoting good health and well-being (Goal 3), ensuring inclusive and quality education for all (Goal 4), and fostering peace, justice, and strong institutions (Goal 16) (https://sustainabledevelopment.un.org/).

International collaborations can help establish measurement tools to be used to measure progress toward meeting these goals. Stakeholders in one country who see evidence that youth in their country are faring more poorly than youth in another country are often motivated to mobilize financial and other resources to support youth development (United Nations, 2022). Investing in PYD has been demonstrated in many contexts to pay a “triple dividend” both in the short term with youth themselves, in the long term when youth grow into adulthood, and in the next generation when youth have children of their own (Camilletti and Banati, 2018). In working toward meeting international goals, it is also important to bear in mind that predictors of PYD may not always be the same across cultures. For example, in countries such as Sweden that have a strong commitment to promoting the rights of young people, positive youth adjustment may be tied to individual agency, self-esteem, self-efficacy, and an internal locus of control (Gurdal et al., 2016). However, in cultural contexts that have more of a focus on the importance of family or community rather than individual rights, positive youth adjustment may be more tied to values such as *familism* in Latinx youth (Stein et al., 2020) or *kapwa* in Filipino youth (Alampay, 2014), which emphasize interconnections between the self and others.

## 5. Conclusions

From a variety of different perspectives and using diverse methods, it is vital to work across generations and cultures to support children, adolescents, and emerging adults as well as to support the many adults (professionals and non-professionals) that have a stake in the development and thriving of young people. As new generations of youth progressively lead and begin to take their place in directing the future of humanity as well as our ecology, it is in the current adult generations' best interest to act with humility (with an openness to hear and learn from youth) as well as authentic generativity toward up-and-coming generations. This Research Topic represents the everyday science that provides more examples and evidence, that illustrate what can be learned when young people are viewed in a holistic light (e.g., Magnusson, 1995) with both their strengths and challenges as the subject of inquiry.

## Author contributions

All authors listed have made a substantial, direct, and intellectual contribution to the work and approved it for publication.

## Footnotes

^1^The articles in this Research Topic were published as soon as the review process concluded, i.e., on a rolling basis. Therefore, the years of publication for the articles described in this section vary.
